# A Metamaterial-Based Compact Planar Monopole Antenna for Wi-Fi and UWB Applications

**DOI:** 10.3390/s19245426

**Published:** 2019-12-09

**Authors:** Adnan Khurshid, Jian Dong, Ronghua Shi

**Affiliations:** School of Computer Science and Engineering, Central South University, Changsha 410083, China; adnan.khurshid1987@gmail.com (A.K.); shirh@csu.edu.cn (R.S.)

**Keywords:** ultrawideband (UWB), dual-band, monopole, composite right left hand (CRLH), left-hand (LH), right-hand (RH), metamaterial (MTM)

## Abstract

Ultrawideband (UWB) antennas are widely used as core devices in high-speed wireless communication. A novel compact UWB monopole antenna with an additional narrow band for Wi-Fi applications comprising a metamaterial (MTM) is proposed in this paper. The antenna has a compact size of 27 × 33 mm^2^ and consists of a V-shaped slot with two rectangular slots in the radiation patch. The inductance and capacitance develop due to the V-shaped slot in the radiation patch. The proposed antenna has −10 dB bandwidths of 3.2 GHz to 14 GHz for UWB and 2.38 GHz to 2.57 GHz for narrowband, corresponding to 144% and 7.66% fractional bandwidths, respectively. The measured gain and efficiency meet the desired values for UWB and Wi-Fi applications. To verify the performance of the antenna, the proposed antenna is fabricated and tested. The simulated and measured results agree well at UWB frequencies and Wi-Fi frequencies, and the antenna can be used as a smart device for portable IoT applications.

## 1. Introduction

Ultrawideband technology has promising prospects in short-range communication systems due to its attractive characteristics such as broad bandwidth, high data-rates, low power spectrum levels, and good radiation performance [1,2]. The UWB frequency range is defined as 3.1–10.6 GHz by the Federal Communications Commission (FCC) [3,4]. UWB antennas play a role in many applications, such as surveillance systems, medical applications, wireless sensor networks, and the Internet of Things (IoT) [5,6,7,8]. However, there are limitations on UWB antennas. These antennas are still large in size, and some frequency bands for short-range communications are not in the UWB spectrum. It would be advantageous to extend the scope of short-range communication systems and to meet commercial and functional requirements, which would require the addition of more frequency bands and a more compact size of UWB antennas for various services. 

A number of approaches have been described in the literature for achieving UWB characteristics. In [9,10,11,12,13,14], the UWB technique was realized through split-ring resonators. Split-ring resonators can be used in many ways, such as for the etching of the patch or ground plane, embedded inside or onto the antenna substrate with different structures, and with a capacitive-loaded strip (CLS). Moreover, the analysis of these structures was always based on several factors, such as wider bandwidth, size, composition, and density. In [15,16,17,18,19], the UWB technique was implemented with a defective ground plane and different shapes of slots or stubs. Although some of these antennas were miniaturized in size, they did not integrate the UWB spectrum with other useful frequency bands for short-range communications.

The concept of UWB technology combined with other short-range communication technologies is a stimulating research topic for enhancing the functionality of radio services. For example, the integration of Wi-Fi with UWB technology can combine Wi-Fi-based localization with UWB-based localization to obtain a hybrid approach that provides sufficient accuracy without the cost of deploying an entire UWB infrastructure [20]. Moreover, in body area network (BAN) communications for gateway services, we can integrate UWB technology with the application of Wi-Fi sensors for effective data transmission in medical applications [21]. In the literature, various methods were reported to achieve UWB bands with other bands, such as using a rectangular-shaped monopole strip [22,23], an elliptical-shaped stub at the high-concentrated current area in the ground plane [24], the strong coupling of complementary sprit-ring resonator (CSRR) or miniaturized resonant capacitors [25,26], and an L-shaped slot and a meander line [27,28,29], for achieving narrowband features in UWB antennas. However, most of these antennas are bulky in size, which is a challenge for the miniaturization of the antenna size. Moreover, in [25], the UWB spectrum was not achieved together with narrow-band. In [26], an antenna was presented with a complex structure to achieve Wi-Fi and UWB bands. Likewise, in [28], an antenna was presented with a bandwidth from 4.5 GHz to 11 GHz which was an incomplete operational range for UWB application. Despite these many designs, we still need low-profile and compact UWB antennas for integration with other frequency bands using simple etching techniques.

In this paper, we proposed a compact monopole antenna for Wi-Fi and UWB applications. The main goal was to maintain the design specifications and antenna coverage for wideband operations as well as to increase the operating bandwidth. This antenna operates well between 2.38–2.57 GHz for narrowband and 3.2–14 GHz for ultrawideband. First, a canonical UWB antenna was designed, and then, by loading a V-shaped resonator, we integrated the narrowband with the UWB in an innovative way. The proposed antenna design, characteristics, and experimental results are discussed in the following sections.

## 2. Antenna Design and Methodology

### 2.1. Antenna Geometry

**Figure 1:**
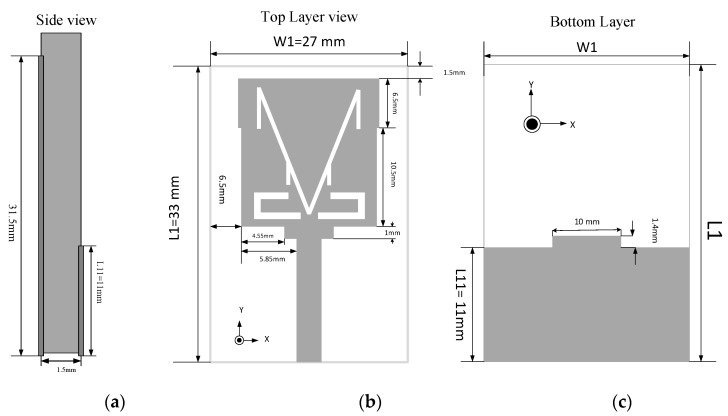
Geometry of the proposed antenna: (**a**) side view; (**b**) top view; (**c**) bottom view.

**Figure 2:**
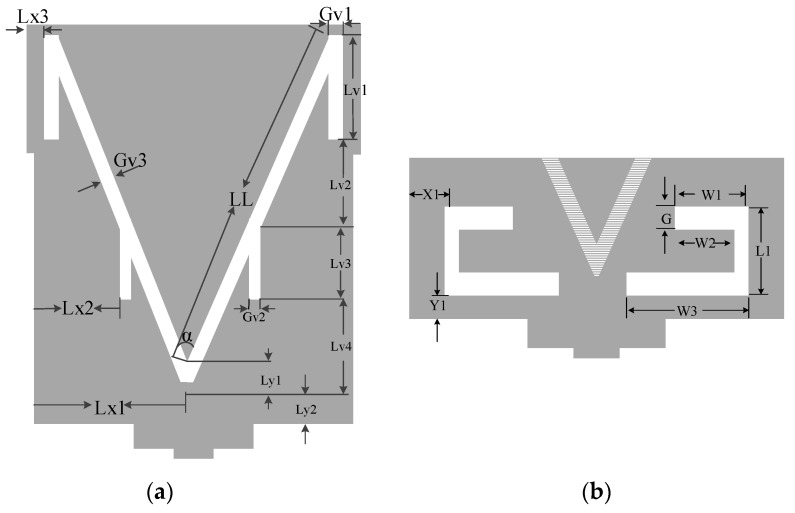
Antenna radiation patch elements: (**a**) V-shaped slot; (**b**) rectangular slot.

The presented antenna, as shown in Figure 1, exhibits dual-band operations with the help of antenna-loaded elements. The monopole antenna contains a 14.64 × 17 mm^2^ radiation patch. The microstrip feedline length is set to 13.5 mm with a width of 2.3 mm to achieve 50 Ω characteristic impedance. The proposed antenna is designed on an FR-4 substrate with a dielectric constant of 4.4, and the tangent loss is δ = 0.02. In Figure 2, the V-slot is defined with an angle of α along with rectangular slots, and the realization of the optimum parameters is discussed in the parametric study. The obtained values of the optimized patch are given in Table 1. There is a protracted area in the patch structure with the feedline, which helps to control the surface current. A stepped structure in the ground plane is used to extend the impedance bandwidth.

### 2.2. Composite Right Left Hand (CRLH) Metamaterial (MTM) Theory

To design a dual-band antenna for the Wi-Fi band and ultrawideband, we introduce inductive and capacitive elements by etching the radiation patch. These reactive elements introduce a phase element between the voltage and current, such as gap capacitance [25] and meander line inductance [29]. To obtain CRLH transmission, we etch the radiation patch, which produces capacitive effects. These effects cause backward waves that travel along the radiation patch to obtain the desired frequency resonant bands and improve the radiation performance along the direction of the radiation patch. The length and width of the slots play a vital role in controlling the magnitudes of the capacitance and inductance. To obtain the properties of the negative refractive index, left-hand effects are observed for a specific range of frequencies. In this novel structure, we observe the strong control of the resonance frequency of the Wi-Fi band and X-band due to CRLH. The CRLH behavior always works as an LC resonator or LC component [30,31]. The inductance and capacitance for series branch resonators and shunt branch resonators are given as follows:

(1) $$\begin{array}{l} L=L_{R}\;,C=C_{R}\;,\mathrm{for}\;\mathrm{series}\;\mathrm{branch}\;\mathrm{resonators},\\ L=L_{L}\;,C=C_{L}\;,\mathrm{for}\;\mathrm{shunt}\;\mathrm{branch}\;\mathrm{resonators}, \end{array}$$

where the subscripts *L* and *R* denote left-handed (LH) and right-handed (RH) possessions, respectively. The resonant frequency of the CRLH components for the *LC* circuit is determined by

(2) $$\omega_{L}=1/\sqrt{L_{L}C_{L}}and\omega_{R}=1/\sqrt{L_{R}C_{R}}$$

where $\omega_{L}$ is the left-hand resonant frequency, and $\omega_{R}$ is the right-hand resonant frequency.

To determine the dispersion relation of a CRLH transmission, we can use the following relationships:

(3) β(ω)=s(ω)ω2LRCR +1ω2LLCL−(LRLL+CRCL) 

(4) $$s(\omega )=\{\begin{array}{c} -1\;\mathrm{if}\;\omega \;<\;\omega_{se}=\min(\begin{array}{cc} \frac{1}{\sqrt{L_{R}C_{L}}}, & \frac{1}{\sqrt{L_{L}C_{R}}}) \end{array}\\ +1\;\mathrm{if}\;\omega \;>\;\omega_{sh}=\max(\begin{array}{cc} \frac{1}{\sqrt{L_{R}C_{L}}}, & \frac{1}{\sqrt{L_{L}C_{R}}}) \end{array} \end{array}$$

(5) $$\omega_{se}=1/\sqrt{L_{R}C_{L}}and\omega_{sh}=1/\sqrt{L_{L}C_{R}}$$

where β(ω) is the phase function of frequency, which can be purely real or purely imaginary depending on whether the radicand is positive or negative; $\omega_{se}$ and $\omega_{sh}$ denote series and shunt-branch resonators, respectively.

The CRLH is increasingly dispersive as the frequency increases because the phase velocity (*v_p_ = ω/β*) becomes increasingly dependent on the resonance frequency, which also shows that low frequencies dominate the LH while high frequencies dominate the RH. From Equation (3), we can identify whether the proposed model follows CRLH properties. In general, the series and shunt resonances of CRLH are different due to unbalanced cases because left-handed transmission supports electromagnetic waves with phase and group velocities that are antiparallel to each other. The apparent backward wave propagation is a key characteristic of CRLH transmission. Due to backward propagation, there is a certain shift in the transition frequency, called the CRLH gap. This gap is due to the left-handed effects. In the designed structure, the V-shaped resonator acts as a shunt capacitor and controls the voltage of the radiation patch to increase the power of the transmission line and reduce the antenna loss. If the bandgap for the left and right-hand region vanishes, then the CRLH region is in a balanced state. In a balanced state, the system impedance *Z_o_*, left-hand impedance *Z_L_*, and right-hand impedance *Z_R_* are equal. Using Equation (6), we can determine the left-hand and right-hand impedances. The characteristic impedance *Z_c_* is always frequency-dependent, as given in Equation (7), and can be related to the material intrinsic impedance $\eta =\sqrt{\mu /\varepsilon }$. 

(6) ΖL=LLCL & ΖR=LRCR

(7) $$Z_{c}=Z_{0}\sqrt{1-\frac{\varepsilon^{2}}{4}\frac{\omega_{L}}{\omega_{R}}}{\;(\mathrm{unbalanced}\;\mathrm{case}\;)\;\&\;Z}_{0}={Ζ}_{L}={Ζ}_{R}(balanced\;case)$$

**Figure 3:**
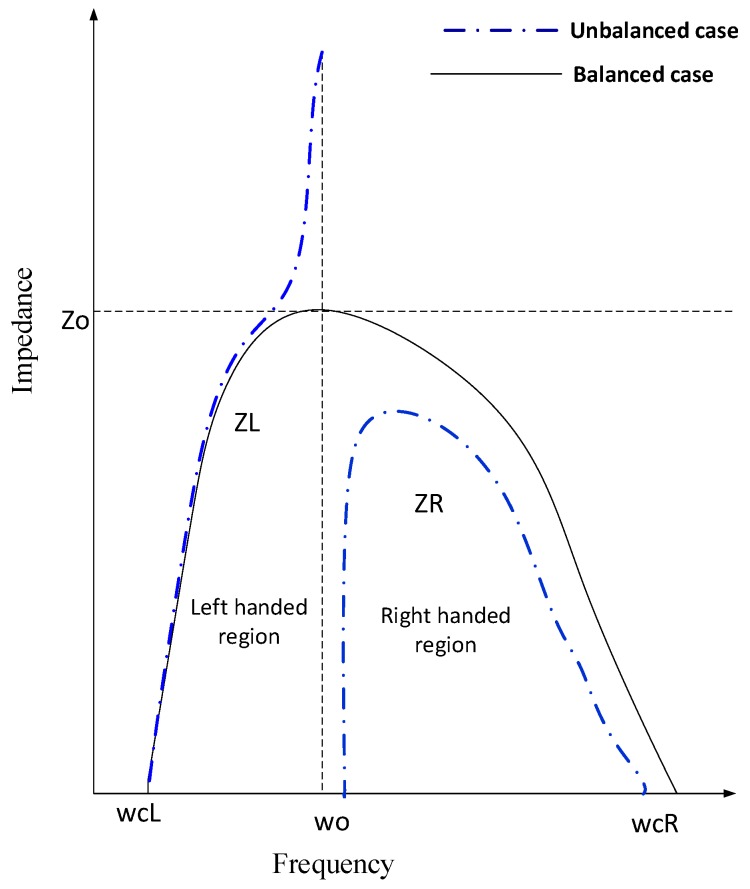
Balanced and unbalanced cases of the composite right left hand (CRLH) region.

The characteristic impedance for balanced and unbalanced CRLH transmission is illustrated in Figure 3. In this figure, *ω_o_* is a transition frequency, as illustrated in Equation (8), where *ω_CL_* is the lower cut off frequency and *ω_CR_* is the higher cut off frequency. The transition frequency separates the left-handed region from the right-handed region. In determining the CRLH region, the permittivity *ε* and permeability *µ* values indicate a negative index for transition frequencies, as given in Equations (9) and (10). If the index is positive, then the material is referred to as right-handed, and if the index is negative, then the material is referred to as left-handed.

(8) $$\omega_{o}=\sqrt{\omega_{R}\omega_{L}}=\frac{1}{\sqrt{L_{R}C_{R}L_{L}C_{L}}}\;$$

(9) $$\mu =\frac{Z}{j\omega }=L_{R}-\frac{1}{\omega^{2}C_{L}}$$

(10) $$\varepsilon =\frac{Y}{j\omega }=C_{R}-\frac{1}{\omega^{2}L_{L}}$$

### 2.3. Antenna Parametric Study

**Figure 4:**
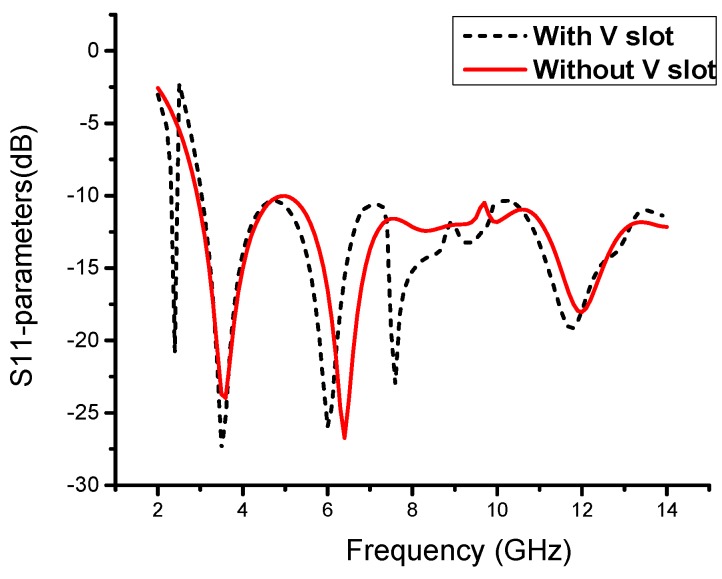
Performance analysis of radiation patch with and without V-slot.

**Figure 5:**
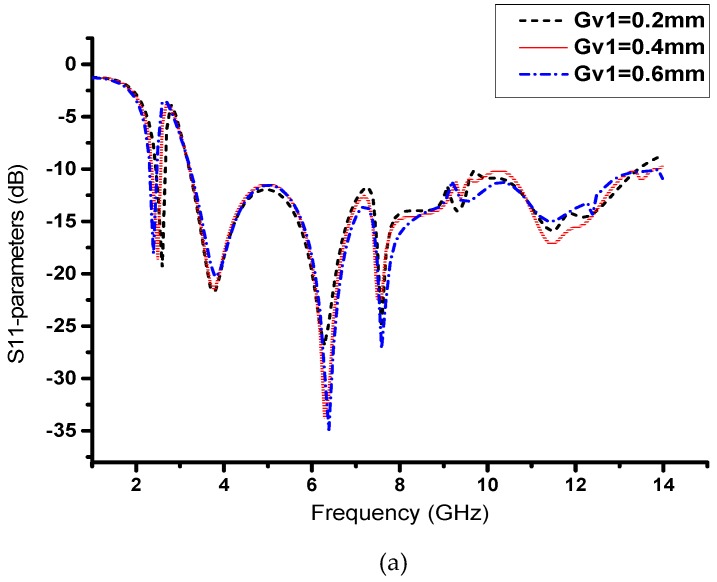

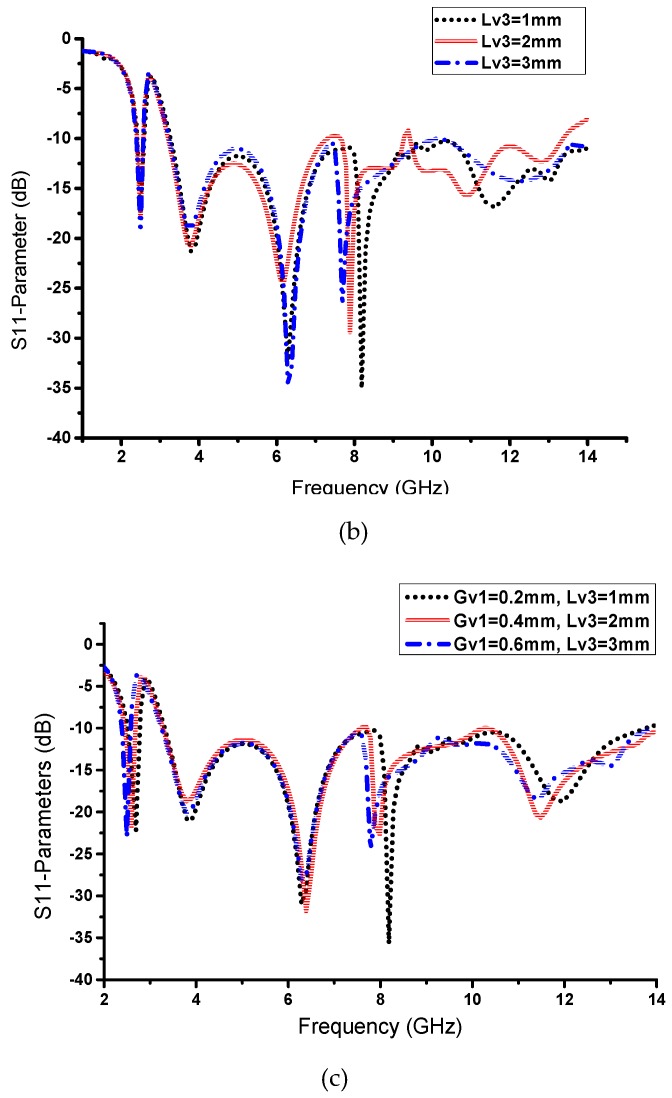
Performance analysis of S_11_ with V-shaped resonant elements: (**a**) variation of Gv1 for 2.48 GHz (Wi-Fi band); (**b**) variation of Lv3 for 7.9 GHz (satellite band); (**c**) variation of Gv1 and Lv3 together.

**Figure 6:**
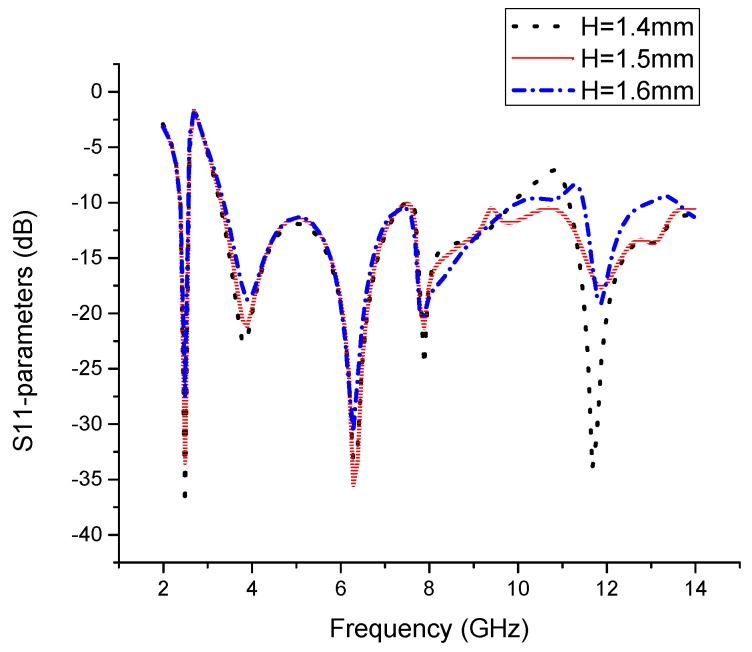
Performance analysis with different substrate heights.

In this novel structure, we introduce a V-shaped resonator that gives us a narrow band for the desired Wi-Fi band, as shown in Figure 4. Furthermore, the V-shaped slot with extended slots shows us the CRLH characteristics, and by varying Gv1, we can adjust our narrow-band resonance frequency from 2.29 GHz to 2.75 GHz, as shown in Figure 5a. We observe that this parameter has a significant effect on the resonance frequency of the narrowband and has a small effect on the UWB spectrum. Likewise, by varying Lv3, we observe the same frequency resonance change at the satellite band from 7.5 to 8.3 GHz, as shown in Figure 5b. These results are observed due to the unbalanced case of CRLH, where the CRLH gap is shifted from the right-hand region to the left-hand region. To verify this resonance deviation, we simulate both parameters together to observe the variation in the resonance frequency. By analyzing both parameters, we achieve a similar resonance response, as shown in Figure 5c. Except for slot parameters, other geometric parameters may also have effects on the antenna matching. Figure 6 show the effect of the substrate height, H, on the return loss when keeping all the other parameters fixed. It can be observed from Figure 6 that the substrate height has a significant effect on the impedance matching of high frequency near 11GHz.

**Figure 7:**
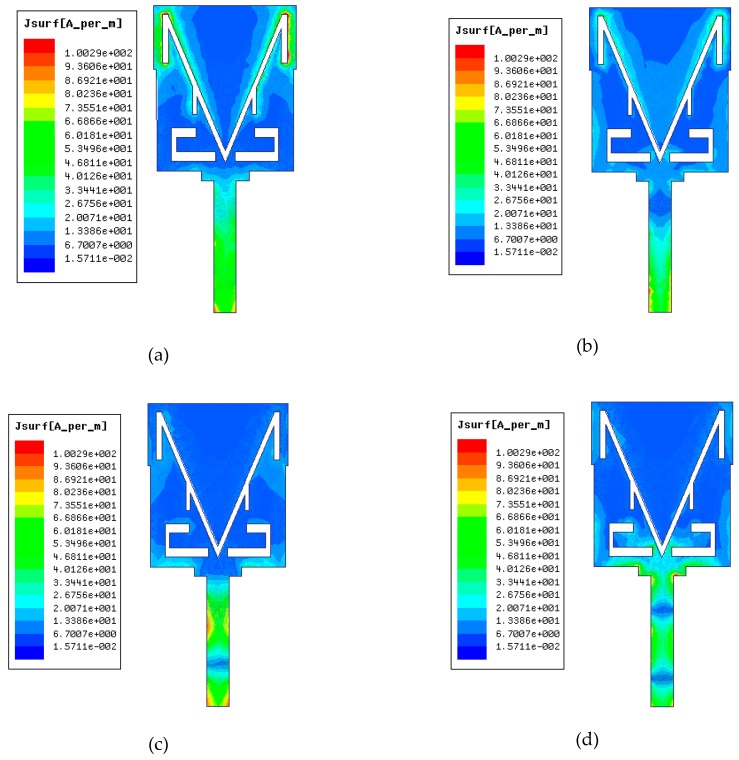
Surface current distributions on the antenna patch at (**a**) 2.48 GHz, (**b**) 3.5 GHz, (**c**) 9 GHz, and (**d**) 11.4 GHz.

The surface current distributions at the frequencies of 2.48 GHz, 3.5 GHz, 9 GHz, and 11.4 GHz are described in Figure 7. The figure clearly shows that the current distributions are different for all four bands. In particular, when the antenna operates at 2.48 GHz, as shown in Figure 7a, the V-slot with an upper extended slot generates the 2.48 GHz frequency band. In Figure 7b, the current is distributed near the V-slot and between the rectangular slots on the radiation patch. In Figure 7c, the current is mainly distributed at the radiation patch corners and the lower part of the radiation patch at 9 GHz, and in Figure 7d, the current is concentrated on the lower part of the radiation patch and around the rectangular slots. These results show that the concentrations of current on the radiation patch are mainly located around the V-slot for lower frequencies, and for higher frequencies, the current concentrations are mainly located at the corners and bottom part of the radiation patch.

## 3. Experimental Results and Discussions

**Figure 8:**
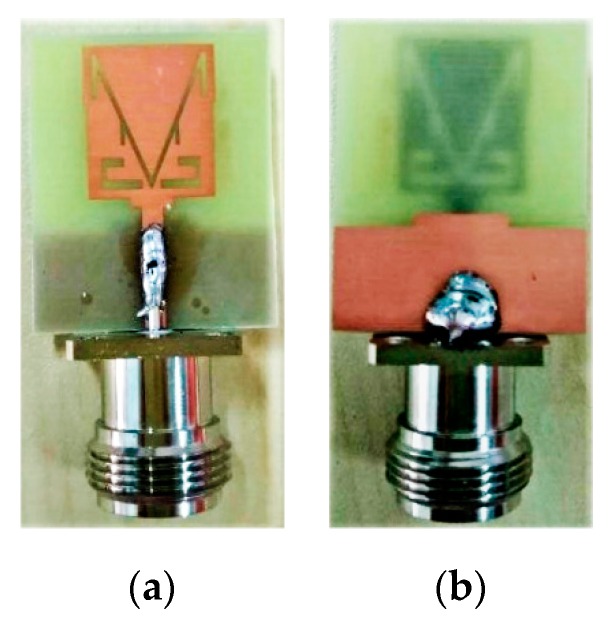
Fabricated proposed antenna prototype, (**a**) front view and (**b**) back view.

**Figure 9:**
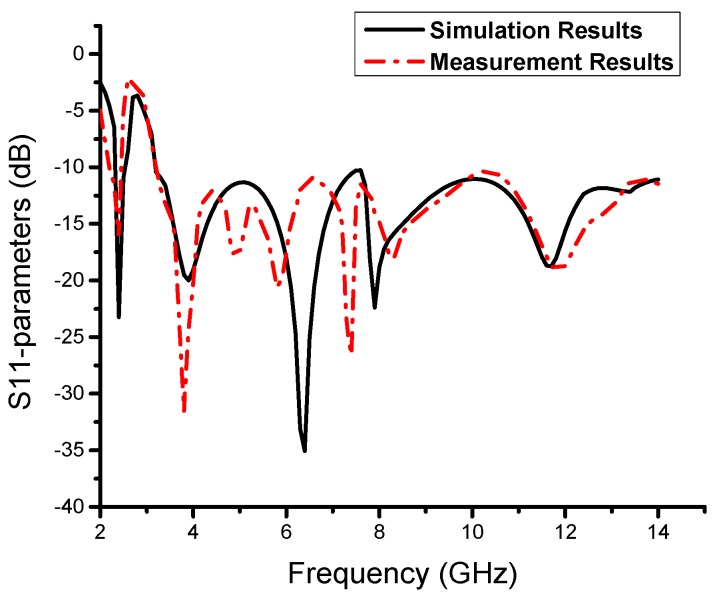
Simulated and measured S_11_ results for the proposed antenna.

**Figure 10:**
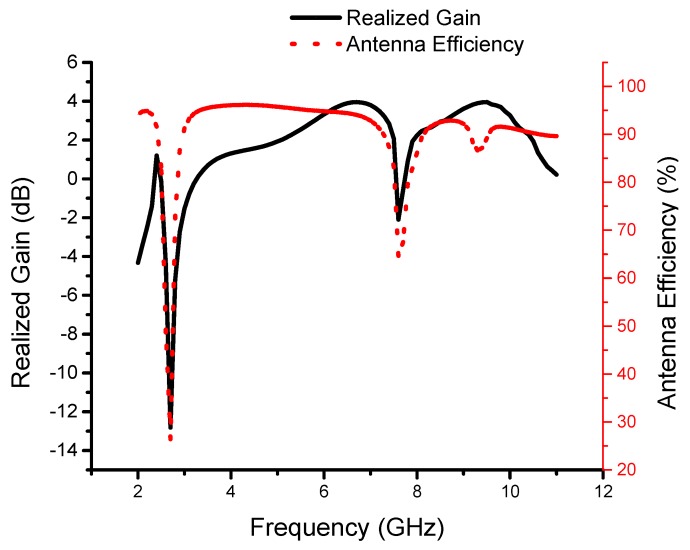
The measured antenna efficiency and realized gain of the proposed ultrawideband (UWB) antenna.

To verify the present antenna model, the proposed monopole MTM antenna is fabricated, as shown in Figure 8. Good agreements between the measurement and simulation S_11_ results are achieved, as shown in Figure 9. The small difference between the measured and simulated results may be caused by the fabrication tolerance and the SMA connector. The resultant impedance bandwidths of less than −10 dB are 2.38 to 2.57 GHz with a fractional bandwidth of 7.66% for narrowband and 3.2 to 14 GHz, which corresponds to a fractional bandwidth of 144%, for the UWB spectrum. We can observe the presence of the CRLH gap in the simulated and measured results. Figure 10 shows the measured realized gain and antenna efficiency. The realized gain is above 1 dB and the antenna efficiency is above 85% at 2.48 GHz, which is sufficient for short-range communication requirements. It is also observed that a large dip in the realized gain and efficiency occurs at 2.7 GHz. From 3.2 GHz to 14 GHz, the antenna gain is almost flat with a peak gain of 3.9 dB except for near the 8 GHz frequency band. Also, from 3.2 GHz to 14 GHz, the antenna efficiency is almost flat at 90% except for near the 8 GHz frequency band.

### 3.1. Radiation Characteristics

**Figure 11:**
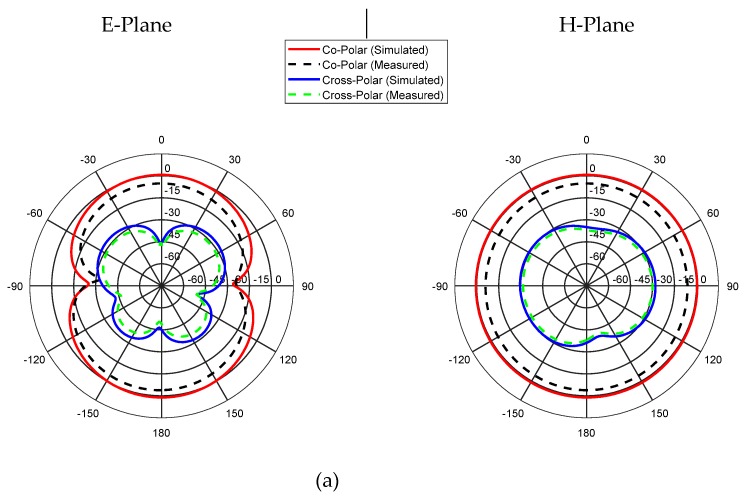

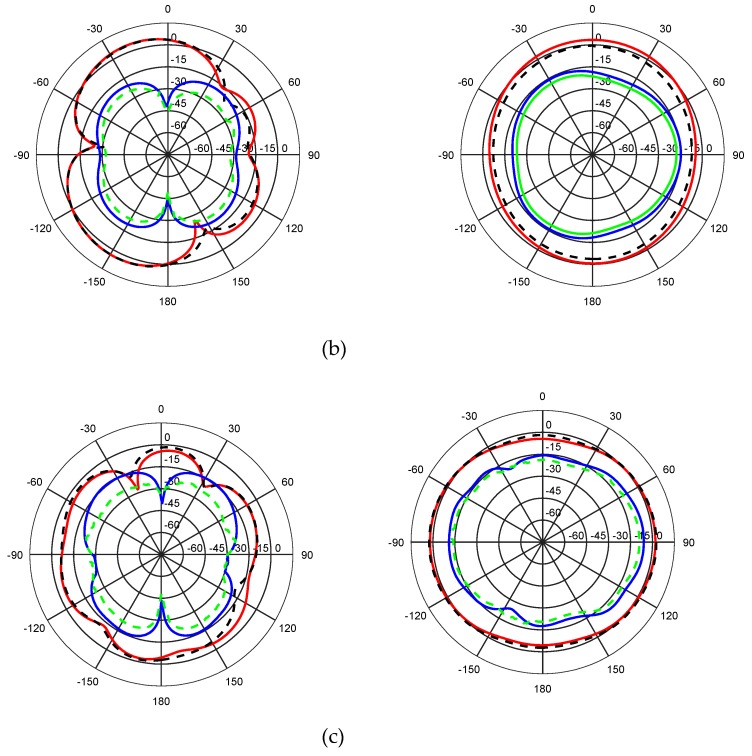
Co- and cross-polar radiation patterns for resonance frequencies of (**a**) 2.48 GHz, (**b**) 7 GHz, (**c**) 11 GHz.

The radiation characteristics of the proposed antenna are shown in Figure 11. The simulated and measured radiation characteristics for the E-plane (yz-plane) and the H-plane (xz-plane) of the antenna are analyzed at three different frequencies; i.e., 2.48, 7, and 11 GHz. There is a good agreement between the measured and simulated results for these frequencies, which indicates that the proposed antenna provides fairly good omnidirectional H-plane patterns and bidirectional E-plane patterns. Additionally, at higher frequencies, the high cross-polarization level is caused by the unequal phase distribution and the significant magnitude of higher-order modes at higher frequencies [23].

### 3.2. Comparison with Other Reported Designs

A comparison of the proposed monopole UWB antenna with other UWB antennas in terms of the design technique, size, peak gain, and operating band is shown in Table 2. It can be observed from the comparison table that our proposed antenna has some obvious advantages such as a simpler structure, a smaller size, and a wider UWB bandwidth with an extra narrowband.

## 4. Conclusions

In this paper, a novel dual-band monopole antenna for Wi-Fi and UWB applications is proposed. A narrowband with the UWB band is introduced to combine different short-range communication applications in a single device. By etching and the inclusion of certain slots in the radiation patch, narrowband together with ultrawideband properties are achieved. The antenna is designed and fabricated, and the simulated response is correlated with the measured results. The measurement results indicate that the designed antenna can operate over 2.38 to 2.57 GHz for narrowband and 3.2 to 14 GHz for ultrawideband. The antenna has a nearly omnidirectional radiation pattern, with more than 90% antenna efficiency. The flat gain can meet short-range communication system requirements, which indicates that this antenna has significant potential for Wi-Fi and UWB applications. This antenna demonstrates that it is a good candidate for short-range applications, as this antenna can be easily integrated with portable devices incorporating multiple communication applications. 

## Figures and Tables

**Table 1:** Design specifications of the proposed antenna structure.

Parameters	Size (mm)	Parameters	Size (mm)	Parameters	Size (mm)
*LL*	15	*Ly1*	1.3	*Ly2*	0.85
*Lv1*	5	*Lv2*	2.52	*Lv3*	2.7
*Lv4*	5	*Lx1*	7	*Lx2*	3.75
*Lx3*	0.9	*Gv1*	0.6	*Gv2*	0.45
*Gv3*	0.5	*α*	46^o^		

**Table 2:** Performance comparison of the proposed antenna with other reviewed antennas. CLS: capacitive loaded strip.

Reference	Technique	Antenna Size (mm) ( λ_o_ = 54 mm)	Antenna peak Gain	Operating band (GHz)	Remarks
[12]	Octagonal patch with complementary sprit-ring resonator (CSRR)	25 × 38 × 1.6 (0.46λ_o_ × 0.7λ_o_ × 0.029λ_o_)	4.6 dB	3.2–9.2	Without narrow band
[13]	Octagonal spiral resonator (OSR), Octagonal SRR (OSRR) and CLS resonators	25 × 25 × 1.6 (0.46λ_o_ × 0.46 λ_o_ × 0.029λ_o_)	3.9 dB	5.2–13.9	Without narrow band
[14]	Rectangular patch	15 × 30 × 1 (0.27λ_o_ × 0.55λ_o_ × 0.018λ_o_)	/	3.1–10	Without narrow band
[15]	Semicircular stepped resonator	58 × 45 × 1.6 (1.07λ_o_ × 0.83λ_o_ × 0.018λ_o_)	10 dB	3.1–10	Large antenna size with a long rectangular shape without Wi-Fi band
[16]	Meshed top patch & patterned ground plane	28 × 32 × 0.79 (0.51λ_o_ × 0.59λ_o_ × 0.014λ_o_)	5.8 dB	3.1–12	Without narrow band
[17]	Hexagonal patch with defected ground plane	20 × 25 × 1.6 (0.37λ_o_ × 0.46λ_o_ × 0.029λ_o_)	5.1 dB	3.09–12.2	Without narrow band
[18]	Cedar tree-shaped	20 × 20 × 1.6 (0.37λ_o_ × 0.37λ_o_ × 0.029λ_o_)	5.5 dB	3–11.7	Without narrow band
[22]	Resonating monopole strip loaded at the center of the patch	52 × 32 × 1.59 (0.96λ_o_ × 0.59λ_o_ × 0.029λ_o_)	7 dB	3.1–11.4 & 2.31-2.59	Large antenna size with long rectangular shape
[24]	Loaded with a quarter-wavelength resonating strip	42 × 24 × 1.6 (0.77λ_o_ × 0.44λ_o_ × 0.029λ_o_)	5 dB	3.1–12 & 2.3–2.5	Large antenna size with long rectangular shape
[26]	Capacitor loaded miniaturized resonator in the ground plane	30 × 31 × 1.5 (0.55λ_o_ × 0.57λ_o_ × 0.027λ_o_)	6dB	3.1–10.6 & 2.4–2.48	Complex structure and relatively small operational bandwidth
[27]	L-shaped stub	46 × 42 × 1 (0.58λ_o_ × 0.77λ_o_ × 0.018λ_o_)	2.81 dB	3.1–10.6 & 2.4–2.48	Large antenna size with long Bluetooth element
[28]	Loaded with long Bluetooth strip	16 × 28 × 1 (0.29λ_o_ × 0.51λ_o_ × 0.018λ_o_)	6 dB	4.5–11 & 2.4–2.48	Incomplete operational UWB spectrum
[29]	Parasitic stub, branch line, and meander line	19 × 28 × 0.8 (0.35λ_o_ × 0.51λ_o_ × 0.014λ_o_)	/	3.1–10.6 & 2.4–2.48	Complex structure and relatively small operational bandwidth
[32]	Loaded with parasitic strip	46 × 20 × 1 (0.85λ_o_ × 0.37λ_o_ × 0.018λ_o_)	4.2 dB	3.1–10.6 & 2.4–2.48	Large antenna size with long rectangular shape
[33]	Strip-line and cutting ground plate	45 × 32 × 1 (0.83λ_o_ × 0.59λ_o_ × 0.018λ_o_)	3.5 dB	3.1–10.6 & 2.4–2.5	Large antenna size with long rectangular shape
**Proposed antenna**	Loaded with V-shaped slot	27 × 33 × 1.5 (0.5λ_o_ × 0.61λ_o_ × 0.027λ_o_)	3.9 dB	3.2–14 & 2.38–2.57	simple and compact size with wider operational bandwidth
